# Large language models as psychometric laboratories? Maybe not yet. Cautionary evidence from synthetic personality data

**DOI:** 10.1002/pcn5.70417

**Published:** 2026-09-21

**Authors:** Matteo Carpi, Conny De Vincenzo

**Affiliations:** ^1^ Department of Human Neuroscience Sapienza University of Rome Rome Italy; ^2^ Department of Education Science Roma Tre University Rome Italy

The rapid growth of large language models (LLMs) has expanded their proposed role in psychological, psychiatric, and personality research beyond text generation, including study design, item generation, data handling, questionnaire administration, and the emulation of respondents with specified personality profiles.^1^, ^2^, ^3^, ^4^, ^5^ These applications are appealing because synthetic samples could support preliminary testing of brief personality measures before costly human data collection. Yet personality fidelity requires more than plausible scores: LLM responses must reproduce the variability and inter‐trait organization found in human personality data.^5^, ^6^, ^7^, ^8^ We therefore conducted an exploratory comparison of human and GPT‐generated responses to a brief personality questionnaire.

The human reference sample comprised 111 Italian university students (mean age = 24.6 ± 6.1 years; 87 women, 22 men, and 2 gender non‐conforming participants) who had completed the best‐performing GPT‐generated Big Five questionnaire from a previous validation study,^4^ which also included comparison with the Ten Item Personality Inventory.^9^ The measure included two items for each Big Five domain: Conscientiousness, Agreeableness, Extraversion, Neuroticism, and Openness. Human responses were contrasted with two synthetic samples (*N* = 150 each) generated using *gpt‐4‐turbo* via the OpenAI API (https://platform.openai.com/). In the synthetic population condition, GPT was repeatedly prompted to complete the questionnaire as an Italian university student. In the synthetic persona condition, GPT responded as a specific individual described by age, sex/gender, study area, housing situation, and working status, with attributes randomly sampled from plausible value sets. We compared item‐ and trait‐level descriptive statistics and Pearson correlations among Big Five traits. Similarity between human and synthetic correlation matrices was quantified by correlating their vectorized off‐diagonal elements.^10^ Full prompts and code are available online in a dedicated GitHub repository (https://github.com/carpcraft/llm_psychometrics), while questionnaire items and scoring rules are fully reported in Table S1 (Supplementary Materials).

Descriptive analyses showed that synthetic responses differed markedly from human responses (Table S2, Supplementary Materials). Human item scores used the response scale broadly, with item‐level standard deviations typically exceeding 1.2. By contrast, the synthetic population condition showed strong variance compression, restricted ranges, and very low dispersion for several items. Persona‐based prompting increased variability, but dispersion remained lower than in the human sample. At the trait level, both synthetic samples showed higher mean Agreeableness, Extraversion, and Openness and lower Conscientiousness, producing homogeneous and normatively positive personality profiles (Figure 1a). Trait variability was lowest in the synthetic population condition and only partly recovered in the persona condition.

**Figure 1:**
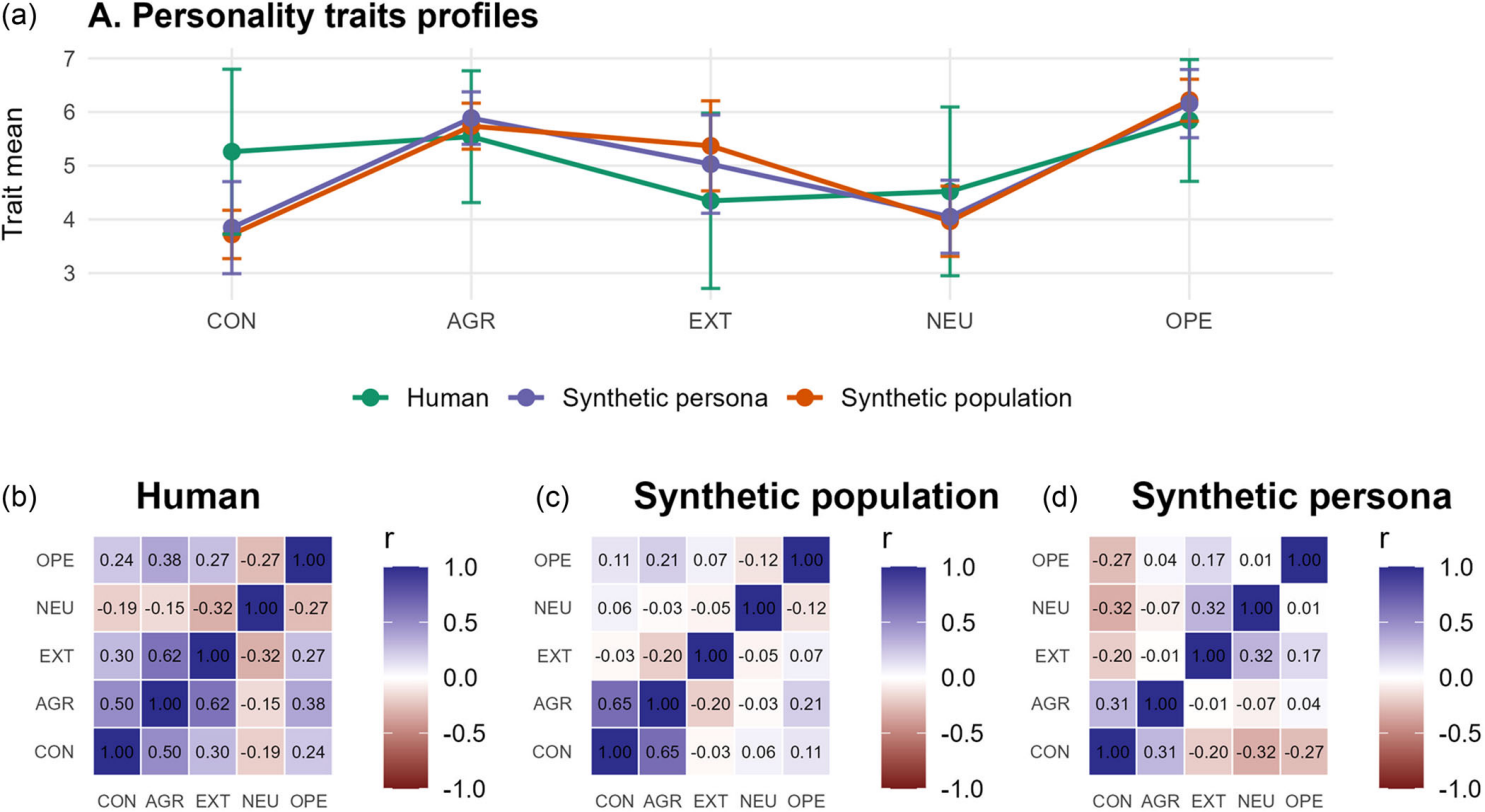
Trait profiles and correlation structures across human and synthetic samples. (a) Shows mean Big Five trait profiles (±standard deviations) for the human sample, the synthetic population sample, and the synthetic persona sample. Panels b–d display Pearson correlation heatmaps among the five Big Five traits for the human sample (b), the synthetic population sample generated without persona constraints (c), and the synthetic persona sample generated using individual‐level demographic prompts (d). AGR, Agreeableness; CON, Conscientiousness; EXT, Extraversion; NEU, Neuroticism; OPE, Openness.

Correlation structures also diverged (Figure 1b–d). The human sample showed a coherent Big Five pattern, including negative associations between Neuroticism and Extraversion and Openness. In the synthetic population condition, most inter‐trait correlations collapsed toward zero, except for an inflated Conscientiousness‐Agreeableness association. The persona condition produced a more articulated but distorted matrix, with sign reversals and large deviations from the human structure, especially for Neuroticism‐related associations. Matrix similarity was modest for the human‐population comparison (*r* = 0.37) and near zero for the human‐persona comparison (*r* = 0.08).

These preliminary findings are specifically cautionary for LLM applications in personality research. Human Big Five profiles describe relatively stable, probabilistic patterns of cognition, affect, motivation, and behavior within an integrated adaptive system,^8^ and their inter‐trait relations are therefore part of personality organization rather than incidental statistical features. By contrast, an LLM generates each response from learned linguistic associations and salient prompt cues, without an enduring individual history that constrains answers across traits. This offers a parsimonious explanation for the observed locally plausible but globally mismatched profiles, suggesting that persona attributes may increase dispersion while cueing demographic stereotypes or trait‐consistent answers independently, thereby distorting inter‐trait associations. This interpretation aligns with personality‐emulation findings showing unrealistically pure GPT factors and limited accommodation of normal trait interdependencies.^5^ This study is limited by its use of a single model family, one brief Italian questionnaire, and a convenience student sample. Nonetheless, the results suggest that LLMs may be useful for item generation or exploratory testing, but should not currently be treated as substitutes for human samples in psychometric validation. Future studies should evaluate longer personality measures, facet‐ and factor‐level structure, criterion validity, and generalization across models, prompts, languages, and populations.

## AUTHOR CONTRIBUTIONS

**Matteo Carpi** and **Conny De Vincenzo:** Conceptualization. **Matteo Carpi** and **Conny De Vincenzo:** Methodology. **Matteo Carpi:** Formal analysis. **Conny De Vincenzo** and **Matteo Carpi:** Investigation. **Conny De Vincenzo** and **Matteo Carpi:** Data curation. **Matteo Carpi** and **Conny De Vincenzo:** Visualization. **Matteo Carpi** and **Conny De Vincenzo:** Writing—original draft. **Matteo Carpi** and **Conny De Vincenzo:** Writing—review and editing.

## CONFLICT OF INTEREST STATEMENT

The authors declare no conflicts of interest.

## ETHICS STATEMENT

The present study involved the secondary analysis of anonymous survey data collected online as part of a previous investigation. No new data collection was performed. In the original study, all participants took part voluntarily and provided informed consent prior to participation through a dedicated anonymous online form. The procedures complied with the principles of the Declaration of Helsinki (2013 revision) and with local institutional ethical standards. According to institutional guidelines, formal ethical approval was not required for this type of research.

## PATIENT CONSENT STATEMENT

Participants provided informed consent for the use and publication of their data in aggregated and anonymous form for scientific purposes, as specified in the original consent procedure.

## CLINICAL TRIAL REGISTRATION

N/A

## Supporting information

Supporting File 1.

## Data Availability

The data supporting the findings of this study are available from the corresponding author upon reasonable request, subject to privacy and ethical constraints. All code used for data generation and analysis is publicly available in a dedicated GitHub repository (https://github.com/carpcraft/llm_psychometrics).
