# Supplementary material for: Large language models as psychometric laboratories? Maybe not yet. Cautionary evidence from synthetic personality data
Source: PCN Rep. 2026 Sep 21;5(3):e70417. doi: 10.1002/pcn5.70417 (PMC13591219; doi:10.1002/pcn5.70417)
Supplement: Supplementary file 1 [file PCN5-5-e70417-s001.docx]

**Large language models as psychometric laboratories? Maybe not yet. Cautionary evidence from synthetic personality data**

**Supplementary Materials**

**Table S1** Item contents of the ten-item GPT-generated personality questionnaire, with original Italian wording reported in square brackets, corresponding Big Five trait, and scoring direction (reverse-scored items flagged). All items were rated on a numerical Likert scale ranging from 1 (“Strongly disagree”; *Fortemente in disaccordo* in Italian) to 7 (“Strongly agree”; *Fortemente d’accordo* in Italian). *Abbreviations:* CON, Conscientiousness; AGR, Agreeableness; EXT, Extraversion; NEU, Neuroticism; OPE, Openness.

| Item | Content | Trait |
| --- | --- | --- |
| Item 1 | I like to have a well-defined routine [*Mi piace avere una routine ben definita*] | CON |
| Item 2 | I prefer to plan ahead rather than improvise [*Preferisco pianificare in anticipo piuttosto che improvvisare*] | CON |
| Item 3 | I consider myself a friendly and warm person [*Mi considero una persona amichevole e calorosa*] | AGR |
| Item 4 | I am always ready to help others [*Sono sempre disposta/o ad aiutare gli altri*] | AGR |
| Item 5 | I enjoy socializing and participating in group events [*Mi piace interagire socialmente e partecipare a eventi di gruppo*] | EXT |
| Item 6 | I feel enthusiastic when I am around people [*Mi sento entusiasta quando sono in mezzo alla gente*] | EXT |
| Item 7 | I am generally calm and do not get upset easily [*Generalmente sono calma/o e non mi agito facilmente*] | NEU (reverse scoring) |
| Item 8 | I don’t usually feel anxious or stressed [*Di solito non mi sento ansiosa/o o stressata/o*] | NEU (reverse scoring) |
| Item 9 | I am always curious to explore new ideas [*Mi incuriosisce sempre esplorare nuove idee*] | OPE |
| Item 10 | I appreciate art and beautiful things in many of their forms [*Apprezzo l’arte e la bellezza in molte delle loro forme*] | OPE |

**Table S2.** Item-level and trait-level means and standard deviations for the three samples (human sample, synthetic population sample, and synthetic persona sample). *Abbreviations:* CON, Conscientiousness; AGR, Agreeableness; EXT, Extraversion; NEU, Neuroticism; OPE, Openness.

| Variable | Human (*N* = 111) | Synthetic population (*N* = 150) | Synthetic persona (*N* = 150) |
| --- | --- | --- | --- |
| Item 1 | 5.2 (1.6) | 4.0 (0.2) | 4.0 (0.6) |
| Item 2 | 5.3 (1.7) | 3.4 (0.9) | 3.7 (1.2) |
| Item 3 | 5.3 (1.4) | 5.5 (0.5) | 5.5 (0.6) |
| Item 4 | 5.8 (1.3) | 6.0 (0.7) | 6.3 (0.6) |
| Item 5 | 4.6 (1.7) | 5.9 (1.0) | 5.4 (1.0) |
| Item 6 | 4.1 (1.8) | 4.9 (0.9) | 4.7 (1.0) |
| Item 7 | 3.8 (1.7) | 4.8 (0.9) | 4.7 (0.9) |
| Item 8 | 3.1 (1.7) | 3.3 (0.6) | 3.2 (0.6) |
| Item 9 | 5.6 (1.4) | 6.4 (0.5) | 6.4 (0.5) |
| Item 10 | 6.1 (1.2) | 6.1 (0.6) | 5.9 (1.00) |
| CON (range 1–7) | 5.3 (1.3) | 3.7 (0.5) | 3.9 (0.9) |
| AGR (range 1–7) | 5.2 (1.0) | 5.7 (0.4) | 5.9 (0.5) |
| EXT (range 1–7) | 3.8 (1.7) | 5.4 (0.8) | 5.0 (0.9) |
| NEU (range 1–7) | 4.0 (1.3) | 4.0 (0.7) | 4.1 (0.7) |
| OPE (range 1–7) | 4.7 (1.1) | 6.2 (0.4) | 6.2 (0.6) |
